# Tapping to drumbeats in an online experiment changes our perception of time and expressiveness

**DOI:** 10.1007/s00426-023-01835-7

**Published:** 2023-06-10

**Authors:** Xinyue Wang, Birgitta Burger, Clemens Wöllner

**Affiliations:** 1 Institute of Systematic Musicology, University of Hamburg, Hamburg, Germany; 2 University of Music Freiburg, Freiburg, Germany

## Abstract

Bodily movements along with music, such as tapping, are not only very frequent, but may also have a profound impact on our perception of time and emotions. The current study adopted an online tapping paradigm to investigate participants’ time experiences and expressiveness judgements when they tapped and did not tap to a series of drumming performances that varied in tempo and rhythmic complexity. Participants were asked to judge durations, passage of time (PoT), and the expressiveness of the performances in two conditions: (1) Observing only, (2) Observing and tapping regularly to the perceived beats. Results show that tapping trials passed subjectively faster and were partially (in slow- and medium-tempo conditions) perceived shorter compared to the observing-only trials. Increases in musical tempo (in tapping trials) and in complexity led to faster PoT, potentially due to distracted attentional resources for the timing task. Participants’ musical training modulated the effects of complexity on the judgments of expressiveness. In addition, increases in tapping speed led to duration overestimation among the less musically trained participants. Taken together, tapping to music may have altered the internal clock speed, affecting the temporal units accumulated in the pacemaker-counter model.

## Introduction

Music offers a unique temporal context that entails varying tempi and complexities, in which we experience time differently. Jonathan Berger (Berger, 2014) has once pointed out that the composition of Franz Schubert’s *String Quintet in C major, D.956* created many temporal illusions—by embedding faster, more complex rhythms in a slow, near motionless musical context, or slow and simple rhythms in a fast, temporally complicated context, Schubert successfully distorted the perceived durations of excerpts in comparison to the clock time.

Many studies have explored the effects of musical attributes on time perception, including tempo (Droit-Volet et al., 2013) and complexity (Bueno et al., 2002). The influences of proactive responses to music such as tapping (Hammerschmidt & Wöllner, 2020; Manning & Schutz, 2013) on time perception have also been widely explored. However, it has not been investigated how individuals perceive time while tapping to music that varies in tempo and complexity. The current study compares perceived time in tapping and no-tapping conditions with a drummer’s performances. We also aim to investigate the perceived expressiveness of the performances in relation to the musical attributes and tapping, to reveal the role it serves in the timing experiences.

### Temporal attributes of music

To investigate the effects music may have on time perception, an understanding of the temporal attributes of music should be established first. The beat provides basic structures to music. It entails isochronous pulses that are subjectively perceived within the individual (Large, 2000), whereas tempo typically refers to the number of perceived beats in a certain period, defined as Beats Per Minute (BPM), yet in fact tempo perception is complex and involves further musical characteristics (London, 2011). Rhythms or rhythmic structures, on the other hand, represent the temporal patterns in which the musical notes are organized with respect to the underlying beat (Large, 2000). Grouping the musical notes by small or large cycles leads to different metrical levels (Burger et al., 2018). A lower metrical level refers to a shorter note length (e.g. adjustment to the eighth note level), while a higher metrical level refers a longer note length (e.g. half note) (Hammerschmidt & Wöllner, 2020). Complexity describes the composition of rhythmic structures such that more complex rhythms temporally encompass more patterns (e.g. polyrhythms) and higher event density (Vuust & Witek, 2014). Rhythmic patterns of various tempi and complexities could lead to differences in the experience of time (Bueno et al., 2002).

### Moving to music affects time perception

Moving to music encompasses a variety of activities, for instance tapping (Polak et al., 2018), walking (Styns et al., 2007), or free whole-body movements (Burger et al., 2018). Among them, tapping has been frequently adopted in studies of sensorimotor synchronization and its effects on time perception, especially in combination with music (Drake et al., 2000; Hammerschmidt & Wöllner, 2020; Snyder & Krumhansl, 2001), as it allows participants to find and to react to beats with small amount of physical efforts.

As previous findings revealed, synchronizing with musical beats as an “organic, effortless, and…spontaneous” (Large, 2000, pp. 527) reaction, such as tapping, could affect the human timing performances to a great extent (Hammerschmidt & Wöllner, 2020; McAuley & Kidd, 1998; Wöllner & Hammerschmidt, 2021). Moreover, it was found that tapping to musical beats increased the accuracy with time keeping tasks (Manning & Schutz, 2013). Durations were perceived to be shorter when tapping to music, whereas time was perceived to pass faster when performing a working memory task (Wöllner & Hammerschmidt, 2021). It should be noted that time perception refers specifically to duration estimation (DE) and passage of time (PoT) (Grondin, 2010), while timing refers to not only passively perceiving time, but also proactively producing temporal structures in various tempi (Honing, 2001).

### Timing mechanisms

Theories and findings of human timing mechanisms, also known as the internal clock model, have shed light on how tapping moderated our timing experiences. The dynamic attending theory (DAT) (Jones & Boltz, 1989; Large & Jones, 1999) supports the central timing model based on Treisman’s (Treisman, 1963) theory, in which the key model is represented as a pacemaker-counter mechanism stands. More specifically, the model hypothesizes an internal clock that emits temporal pulses, records the accumulated pulses within the target period, and compares the recorded pulses to the ones of a reference duration before coming to judgments. Based on the pacemaker-counter model, the DAT postulates that the emission of internal temporal pulses can be synchronised with external rhythms, also known as the temporal entrainment effect (McAuley & Jones, 2003). Attending to faster stimuli leads to increases in the internal temporal pulses, and consequently dilation of the perceived duration and reduced passage of time (PoT). The effect has been validated as robust and widely present across a variety of sensory modalities (Wang & Wöllner, 2019), thus validating tempo as a key predictor of the perceived duration and PoT. When tapping to higher metrical levels, participants attended to larger temporal units and consequently judged perceived durations to be shorter and PoT to be faster (Hammerschmidt & Wöllner, 2020). Thus, by tapping to the music, individuals explicitly synchronize the internal clock speed to the underlying temporal pulses of the external rhythms and experience changes in the perceived time.

### Effects of expressiveness and complexity

The emotional expressiveness may also mediate how individuals perceive the passing of time in relation to the internal clock speed. Fast movements were perceived to be more expressive (Allingham et al., 2021), indicating that fast stimuli positively predicted the perceived expressiveness. This finding with visual movements is in line with auditory evidence where an association between tempo of the music and expressiveness was found (Fernández-Sotos et al., 2016). Considering faster clock speed associated with higher expressiveness, perceiving expressive stimuli could also lead to overestimation of the duration and slower passage of time.

Complexity is another factor that has been found to affect temporal processing. Moderately complex stimuli containing higher event density within a fixed period of time were perceived to be longer than simple and/or highly complex ones (Aubry et al., 2008; Bueno et al., 2002; Hogan, 1975). According to the pacemaker-counter mechanism (Treisman, 1963), more temporal units accumulated in the counter device could lead to duration estimation, as the internal clock speed synchronises with frequent event changes (high number of segmentations) with the complex stimuli (Fraisse, 1978). On the other hand, Mate and colleagues (Mate et al., 2009) proposed that highly complex stimuli required more resources in the working memory, thus could be judged to be longer in comparison with the reference duration stored in the pacemaker-counter device. The effect was also found with musical stimuli, as participants overestimated the durations as they listened to 90-s excerpts of a rhythmically simple versus a complex symphony (Bueno et al., 2002).

### Effects of music training

A key influence on the perception of time is the perceivers’ musical expertise. Musicians are capable of more accurate duration estimation (Panagiotidi & Samartzi, 2012; Rammsayer & Altenmüller, 2006), sensorimotor synchronization (Drake et al., 2000; Repp, 2010), temporal phase detection (Manning & Schutz, 2016), and higher synchronization flexibility (Scheurich et al., 2018) than non-musicians. 12- to 15-year-old students who received at least two years of musical training estimated durations of musical excerpts more accurately than those who did not (Panagiotidi & Samartzi, 2012). Moreover, modality-specific evidence supports the view that musically trained individuals exhibited more stable and more accurate sensorimotor synchronization with auditory rhythms than the untrained group, due to their extensive training in tasks such as collaborative music making that frequently involve time keeping (Repp, 2010; Repp & Doggett, 2007). Altogether, the findings suggest that musical training equipped individuals with higher accuracy and sensitivity in temporal processing.

### Online tapping paradigms

In past studies, researchers have mainly adopted in-lab setting for the consistency of environment and standardization of procedure. Experiments were run with in-lab tapping devices such as Yamaha piano keyboard (Snyder & Krumhansl, 2001), a BopPad touch pad (Hammerschmidt & Wöllner, 2020), or the space bar of the experiment computer (London et al., 2019). The recent tapping apparatus has shifted to online platforms, for instance, the Rhythm ExPeriment Platform (REPP) (Anglada-Tort et al., 2022) and web-based tapping applications (Hammerschmidt et al., 2021a). In response to the current demands, we aimed to develop an easy and direct way to implement a tapping study using an existing online survey platform SoSci Survey (Leiner, 2019).

### Aims

In the current study, we aimed to investigate whether judgments on Duration Estimation (DE), Passage of Time (PoT), and Expressiveness are affected when tapping to audio-visual stimuli of varying tempi and rhythmic complexities compared to when not tapping. In addition, we explored the effects of musical training on the perceptual ratings, as measured by the Gold-MSI (Müllensiefen et al., 2014). To further inspect possible effects of tapping, tapping speed and stability were also examined. We hypothesized that:

1. Tapping with the performance leads to faster PoT and shorter DE compared to the no-tapping conditions.
2. Due to the higher event density, fast performances are assumed to be perceived as longer, to pass more slowly, and be more expressive than slow ones. Similarly, complex rhythms are expected to be perceived as longer, pass more slowly, and be more expressive than simple rhythms.
3. Higher musical training is expected to lead to more accurate DE and PoT judgments, as past study found higher accuracy with musically trained groups (Nguyen et al., 2022).
4. Fast tapping as well as high tapping stability are predicted to be linked to duration overestimation and slower PoT, as they indicate faster and more stable internal clock speed, while slow tapping and low stability should lead to duration underestimation and faster PoT.

## Method

### Participants

A total of 109 participants were recruited for the online experiment (61.5% were females; *M*_age_ = 26, SD_age_ = 7.04). Over half of the participants (56.6%) have completed higher education (Bachelor, Master, and Ph.D. degrees). Participants were of a wide variety of nationalities, mainly Europeans (49.54%) and Africans (41.84%). Based on the summed scores of five items from the Goldsmith Music Sophistication Index (part of the Gold MSI factor 3 “Music Training”, Müllensiefen et al., 2014; see Supporting Information I), the participants’ music training scored a mean of 0.25 (SD_MusicTraining_ = 0.05) after being normalized, ranging from 0 (no music training) to 1 (highly trained). Thirty-two participants had not received any formal music training, while 77 had received some type of formal training. Participants’ original musical training score, based on selected items, ranged from 5 (no training) to 34 (highly trained), including the years of training and hours of daily practice, among other variables. The musical training score was normalized for subsequent analyses with its range as described above.

Participants were recruited via the survey platform Prolific (https://prolific.co/) and university classes to participate in the online experiment on SoSci Survey (https://www.soscisurvey.de) (Leiner, 2019). The study was approved by the Ethics Committee at the Faculty of Humanities, University of Hamburg. All participation were consented. Each participant was either compensated by an hourly rate of €8.85/hour or course credits. On average, it took participants 20 min to complete the study.

### Stimuli

**Fig. 1:**
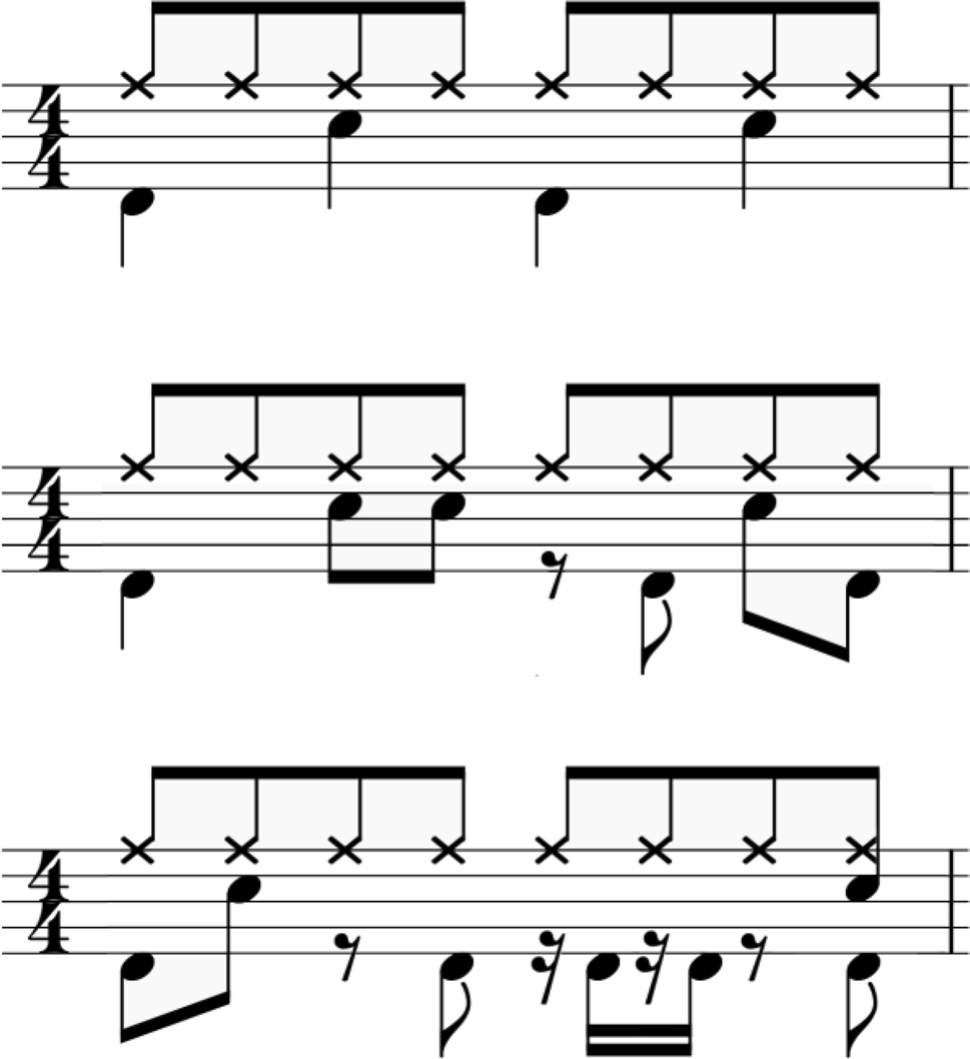
Depiction of three rhythmic complexities from simple (top), medium (middle), to complex (down) condition. The lower filled notes indicate the kick, the higher filled notes the snare, and the cross noted the hihat

**Fig. 2:**
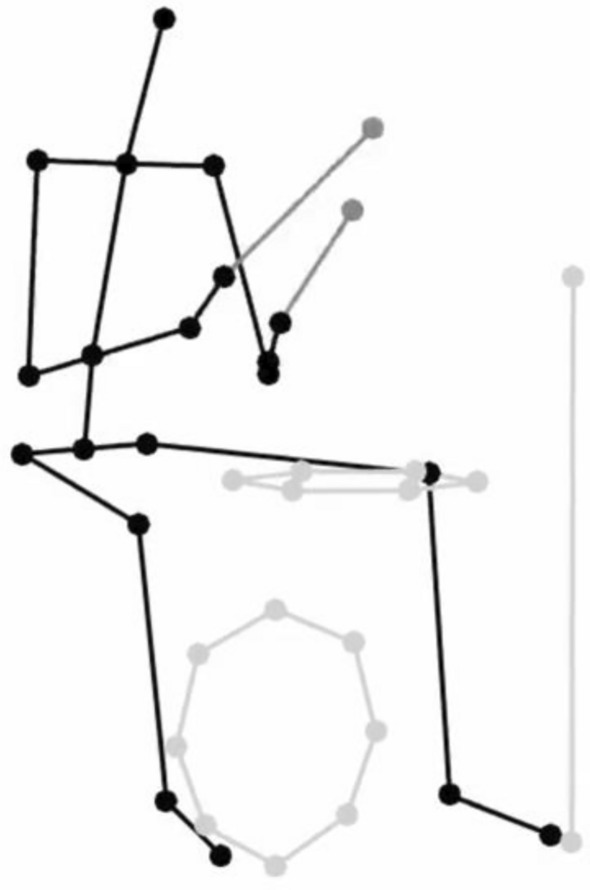
Depiction of the biological motion of the drummer’s performance as the visual stimuli. The stimuli can be found on Zenodo (10.5281/zenodo.7987993)

The experiment stimuli consisted of 9 different audio-visual presentations, in which a drummer (male, 27 year-old, classically trained for over 20 years) performed three rhythms in three tempi (60, 110, and 160 BPM) and three levels of complexities (simple, medium, complex, see Fig. 1). The movements were recorded via an eleven-camera motion-capture system (Qualisys Oqus 700) at a framerate of 200 frames per second, depicting the performer, drum sticks, and the drumming instruments (kick, snare, hihat) using reflective markers. Animations were created using MoCap toolbox (Burger & Toiviainen, 2013) in MATLAB (black stick figure on a white background, see Fig. 2).

Each experiment stimulus started by showing a fixation cross in the centre of a dark background lasting one second, followed by a drumming performance lasting 15 s. In addition, 4 catch trials of different lengths (2 of 8 s, 2 of 30 s) were included that did not enter any analysis, as their sole purpose was to investigate whether participants attended to the durations of the stimuli.

### Procedure

Participants were required to complete the tasks in a quiet environment on the computer using headphones. They were given the opportunity to adjust their sound level with a sample music excerpt before the start. Participants were then asked to tap the spacebar regularly for one minute upon instructions on the screen to measure their pre-test spontaneous motor tapping (SMT), and then rate how excited (emotional arousal) they felt by moving the cursor on a scale. Following this, the experiment part started containing two blocks, the first one required observing and rating the drumming animations, while the second block required observing and simultaneously tapping to the drumming animations and rating them. The order of the blocks was not randomized because participants should complete the observing-only trials having no knowledge of motor involvement. The order of the stimuli within each block was randomized to avoid any effect of order. To introduce the first (i.e., no-tapping) task, a test trial was presented with participants being asked to watch the animation first and then type their estimation of the stimulus duration. They also typed their estimation of the duration in seconds, and used a slider on a scale from 1 to 101 to indicate how fast they personally felt time had passed and how expressive the performance was. The first block of 11 randomised no-tapping trials (including two catch trials) commenced after the test trial. The participants were free to take a break between the first and second block.

Before the tapping block started, participants were informed that they should tap with the performance in a regular, even, and non-rhythmic manner on the spacebar. Video examples of correct and incorrect tapping, performed by one of the authors of this study, were presented to instruct and guide the participants (material and data are available on Zenodo. For material, it can be found at: 10.5281/zenodo.7987993, for data, it can be found at: 10.5281/zenodo.7988013). Participants were again presented with a test trial, followed by the second block. During each stimulus presentation, the phrase “Please tap to the performance” was always displayed as a reminder. After the second block, participants were asked to complete a post-test SMT of the same procedure as the pre-test SMT and to rate their emotional arousal level again. They were also required to fill in a short questionnaire of their music training and demographic information. During the process, participants were instructed not to look at any clocks or to count the time.

### Analyses

Two streams of analyses were conducted: (1) To examine effects of tapping versus no-tapping in the context of performance and music training differences, perceptual judgements of the tapping and no-tapping trials were predicted by the stimulus characteristics and music training. (2) To investigate the impact of tapping behaviour more specifically, perceptual judgements of the tapping trials were predicted by tapping speed, tapping stability, stimulus characteristics, and music training.

After checking that the SoSci Survey tapping data allowed reliable post-processing, taps between the third and the last one were extracted for analyses purposes. This was done to reduce participant instability, as they needed a few taps to get into the tempo of the trial. Subsequently, outlier detection was conducted before the commencement of analyses. Data of participants (*N* = 12) who failed to tap in less than 10 of the 11 trials in each block were completely removed from the dataset of perceptual ratings, tapping recordings, and demographics, due to failure to understand the nature of the tasks. From a pool of 29,924 taps, we eliminated outliers based on the following criteria: (1) Less than 3 taps in one trial, (2) inter-tap intervals (ITIs, temporal distance between two consecutive taps) longer than the 25% upper threshold of a whole note at the tempo, which might be due to absence of attention and indicates disruption of the task in the trial, (3) Taps of repetitive timestamps, in other words ITIs consecutively (*N* > 1) equal to 0, that might be due to system failures (*N*_*Taps*_ = 117), (4) ITIs lower than 70 ms, which suggested tapping twice by mistake in a very short period (*N*_*Taps*_ = 51), (5) ITIs longer than 5 SDs from the mean averaged from all ITIs per participant per condition/trial except for the maximum ITI (*N*_*Taps*_ = 116). Trials (*N*_*Taps*_ = 4) that fit the first and second criterion were completely removed, while taps that fit the other criteria were removed from perspective trials keeping the remainder of the trial. Data of participants whose tapping trials after outlier exclusion were less than 10 out of 11 were not eliminated.

For the first stream of analysis (influence of tapping vs. no-tapping), general linear mixed models (LMMs) were adopted to answer whether (1) tapping, (2) tempo, (3) complexity, (4) musical training, also referred to training in the following, and their two-way interactions (fixed effects) affected participants’ DE, PoT, and Expressiveness judgments. Thus, independent variables include tapping (yes/no), tempo (3 levels), complexity (3 levels), and training (based on the Gold-MSI; Müllensiefen et al., 2014). To select the model of the highest goodness of fit for each dependent variable, maximum likelihood ratio (MLR) tests were conducted (see Table 2). In the MLR tests, predictors were added one after another from the baseline model, in which only the random effects were present. Variance of participants and of conditions were considered as random effects. Models with the lowest Akaike Information Criterion (AIC), while adding significantly to the previous model were considered the final models. Therefore, for this line of models, each LMM might entail a different number of predictors. Post-hoc analyses with Tukey correction were conducted to follow up significant main and interaction effects.

$$Perceptual\, judgments=\alpha +{\beta }_{Tap}{X}_{Tap}+{\beta }_{Tempo}{X}_{Tempo}+{\beta }_{Comp}{X}_{Comp}+{\beta }_{MT}{X}_{MT}$$

$$+\,{\beta }_{Tap:Tempo}{X}_{Tap}{X}_{Tempo} +{\beta }_{Tap:Comp}{X}_{Tap}{X}_{Comp} +{\beta }_{Tap:MT}{X}_{Tap}{X}_{MT}$$

$$+\,{\beta }_{Tempo:Comp}{X}_{Tempo}{X}_{Comp }+{\beta }_{Tempo:MT}{X}_{Tempo}{X}_{MT}$$

$$+\,{\beta }_{Comp:MT}{X}_{Comp}{X}_{MT}+\left(1|Participant\right) +\left(1|Condition\right)$$

In this equation, perceptual judgments stand for DE, PoT, or Expressiveness, $\alpha$ stands for the fixed intercept, while *β* represents the betas of the fixed effects. Comp = Complexity, MT = Music training (normalized scores). It should be noted that components of the equation vary based on MLR test results (see Table 1).

**Table 1:** Summary of the mixed linear model analysis for all trials based on the MLR results

DV	Variable	B	SE B	*t*	*p*
Duration estimation	${\beta }_{Tempo:Tap}$	− 0.07	0.02	− 3.18	0.002**
Passage of time	${\beta }_{Comp}$	0.40	0.14	2.81	0.016*
	${\beta }_{Tap}$	0.33	0.15	2.18	0.049*
	${\beta }_{MT}$	− 0.24	0.09	− 2.68	0.007**
	${\beta }_{Tempo:MT}$	0.06	0.02	2.84	0.005**
	${\beta }_{Tap:MT}$	0.08	0.06	2.35	0.019*
Expressiveness	${\beta }_{Tempo}$	− 0.13	0.01	− 9.76	< 0.001***
	${\beta }_{MT}$	− 0.25	0.11	− 2.34	0.019*
	${\beta }_{Tap:MT}$	0.11	0.04	2.89	0.004**
	${\beta }_{Comp:MT}$	0.05	0.02	2.09	0.037*

For the full table, please refer to Table S2. Tapping was coded as 1 (no-tapping) or 2 (tapping). Tempo was coded as 3 (slow), 2 (medium), and 1 (fast). Complexity was coded as 1 (simple), 2 (medium), and 3 (complex)

*Comp* complexity, *Tap* presence of tapping, *MT* normalized music training scores

***p < 0.001, **p < 0.01, *p < 0.05

For the second stream of analyses (influence of tapping speed and stability), LMMs were adopted to answer the research question of whether (1) tapping speed, (2) tapping stability, (3) tempo, (4) complexity, and (5) participants’ music training as well as their two-way interactions affected participants’ DE, PoT, and Expressiveness judgments within the tapping trials. This line of models was intended to examine the contributions of tapping speed and stability, as well as their interactions with other factors. In this regard, MLR tests were not adopted to examine the goodness of fit. Variance from participants and conditions were included as random effects. Post-hoc analyses with Tukey correction were conducted to follow up significant main and interaction effects. The analyses were performed in R (Version 3.5.3; R Core Team, 2019) using the package lme4 (Bates et al., 2015).

$$Perceptual \,judgments=\alpha +{\beta }_{ITI}{X}_{ITI}+{\beta }_{ITIcv}{X}_{ITIcv}+{\beta }_{Tempo}{X}_{Tempo}+{\beta }_{Comp}{X}_{Comp}+{\beta }_{MT}{X}_{MT}$$

$$+\,{\beta }_{ITI:Tempo}{X}_{ITI}{X}_{Tempo} +{\beta }_{ITI:Comp}{X}_{ITI}{X}_{Comp} +{\beta }_{ITI:MT}{X}_{ITI}{X}_{MT}$$

$$+\,{\beta }_{ITIcv:Tempo}{X}_{ITIcv}{X}_{Tempo} +{\beta }_{ITIcv:Comp}{X}_{ITIcv}{X}_{Comp} +{\beta }_{ITIcv:MT}{X}_{ITIcv}{X}_{MT}$$

$$+\,{\beta }_{ITI:ITIcv}{X}_{ITI}{X}_{ITIcv}+\left(1|Participant\right) + \left(1|Condition\right)$$

In this equation, perceptual judgments stand for DE, PoT, or expressiveness, $\alpha$ stands for the fixed intercept, while *β* represent the betas of the fixed effects. MT = Music training (normalized scores), Comp = Complexity, ITI = mean inter-tap intervals (tapping speed), ITIcv = Coefficient of variation for inter-tap intervals (tapping stability).

**Fig. 3:**
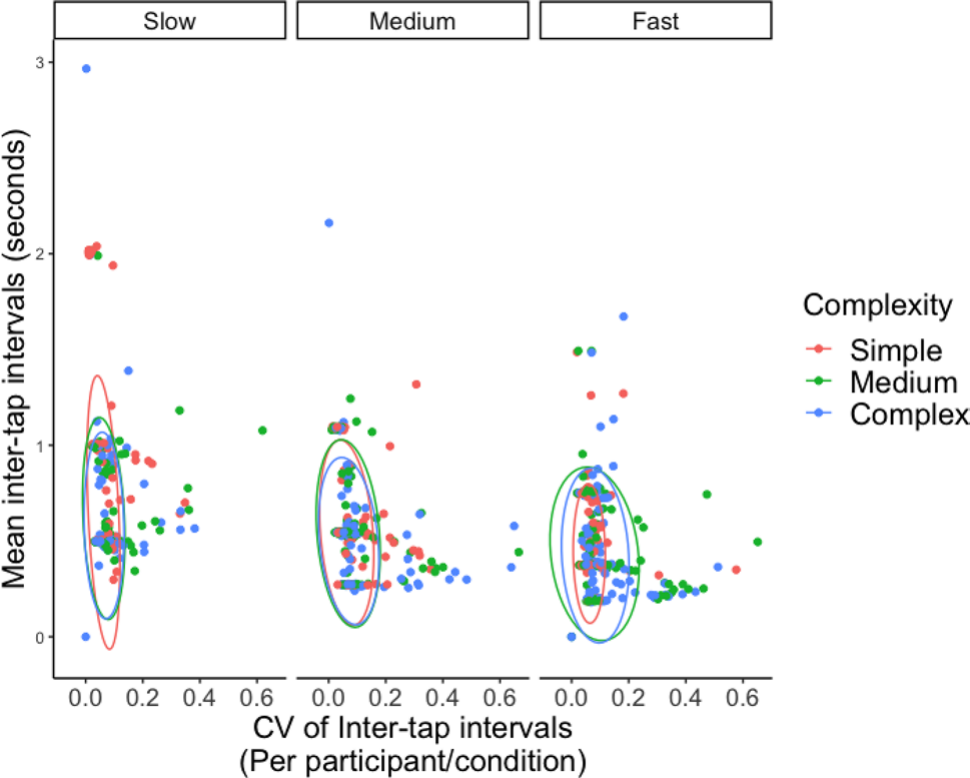
Scatter plots of the mean I (y-axis) and CVs of ITI (x-axis) clustered by rhythmic complexity, faceted by tempo

For this stream of models, only the ratings for the tapping trials were included as well as participants’ tapping behaviour. Tapping data were transformed into two variables: (1) Tapping speed: Average ITI per participant per condition, the higher the it is, the slower the tapping tempo. (2) Tapping stability: Coefficient of variation (CV) of it is per participant per condition, which is the ratio between standard deviation and mean (standard deviation (sd)/mean). The higher the CV of ITI, the more unstable the taps. The descriptive scatter plots (see Fig. 3) show that participants’ tapping behaviours were mainly isochronous, consistent, and clustered around different tempo and complexity conditions.

For both lines of analyses, dependent variables include DE, PoT, and Expressiveness.

- DE: Participants’ estimation of the time passed in seconds.
- PoT: The ratings from a 1 (slowest) to 101 (fastest) scale was normalized across the full range of the scale (x − min(x))/(max(x) − min(x))). Minimum was 1, while maximum was 101.
- Expressiveness: The ratings from a 1 (not at all) to 101 (very much) scale was normalized across the full range of the scale (x − min(x))/(max(x) − min(x))). Minimum was 1, while maximum was 101.

## Results

LMMs were conducted to examine participants’ judgements in DE, PoT, and Expressiveness in tapping versus no-tapping trials. The effects of training, tempo, and complexity as well as their interactions with tapping were included in the models.

### Influence of tapping versus no-tapping

#### Duration estimation

**Fig. 4:**
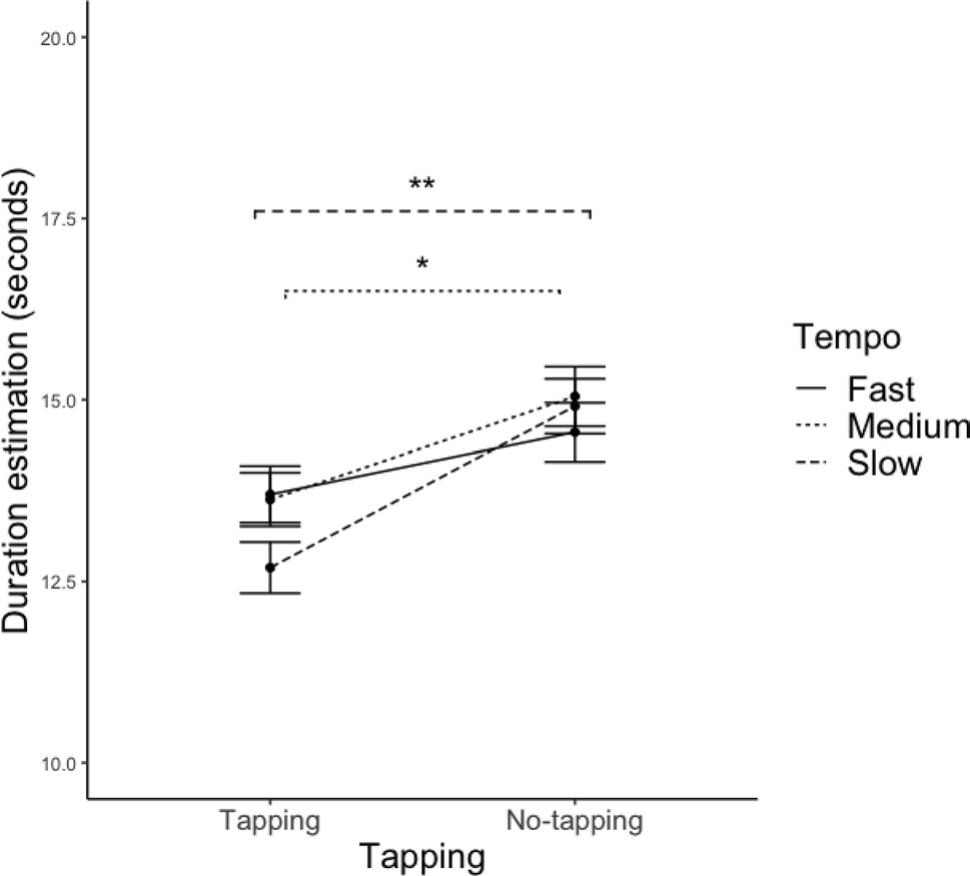
Line plot of the interaction effect between tempo and tapping on Duration Estimation. The whiskers represent standard errors. **p < 0.01, *p < 0.05. Significance indicators are of the same line type as the corresponding groups (i.e. tempo)

No significant main effects of tempo, rhythmic complexity, tapping, and training were found, while there was a significant interaction effect between tempo and tapping (see Table 1, for the full table, please refer to Table S2). Post-hoc comparison with Tukey correction suggested that, in this interaction, the effect of tapping was significant for the slow and medium tempi: No-tapping trials were perceived to last longer than the tapping trials (see Table 2, Fig. 4, for the full table, please refer to Table S3). No significant differences were found in the fast condition.

**Table 2:** Summary of the post-hoc comparison with Tukey correction based on the significant interactions from Table 1

DV	IV1	IV2	Differences of estimate	SE of difference	*t*	*p*
Duration estimation	Slow	T-NT	2.22	0.30	7.29	0.011*
	Medium	T-NT	1.42	0.30	4.65	0.053*
Expressiveness	High MT	T-NT	− 0.04	0.02	− 2.84	0.043*
	High MT	Simple-complex	− 0.02	0.02	− 3.57	0.018*

For the full table, please refer to Table S3. Coding of the variables is consistent with the caption of Table 1

*T* tapping, *NT* no-tapping, *MT* music training

***p < .001, **p < .01, *p < .05

### Passage of time

Significant main effects of complexity, tapping, and training were found, whereas no significant effect of tempo was present. More complex rhythms are related to faster PoT. However, post-hoc comparisons with Tukey corrections suggested no significant differences among the three levels of complexity (see Table 3). The model revealed significant interaction effects between training and tempo as well as between training and tapping (see Table 1, for the full table, please refer to Table S2). Post-hoc comparisons with Tukey correction suggested that the effect of tempo did not differ significantly between high and low musical training. Similarly, the effect of tapping did not differ significantly by levels of musical training (see Table 2).

**Table 3:** Summary of the post-hoc comparison with Tukey correction based on significant main effects from Table 1

DV	Condition1	Condition2	Differences of estimate	SE of difference	t	p
Passage of time	Simple	Complex	− 0.13	0.07	− 1.72	0.276
	Simple	Medium	− 0.13	0.07	− 1.72	0.268
	Medium	Complex	0.001	0.07	0.01	0.999
Expressiveness	Slow	Fast	− 0.25	0.02	16.08	< 0.001***
	Slow	Medium	− 0.18	0.02	11.55	< 0.001***
	Medium	Fast	− 0.07	0.02	4.54	0.002**

For coding of variables, see Table 1

*T* tapping, *NT* no-tapping

***p < 0.001, **p < 0.01, *p < 0.05

### Expressiveness

**Fig. 5:**
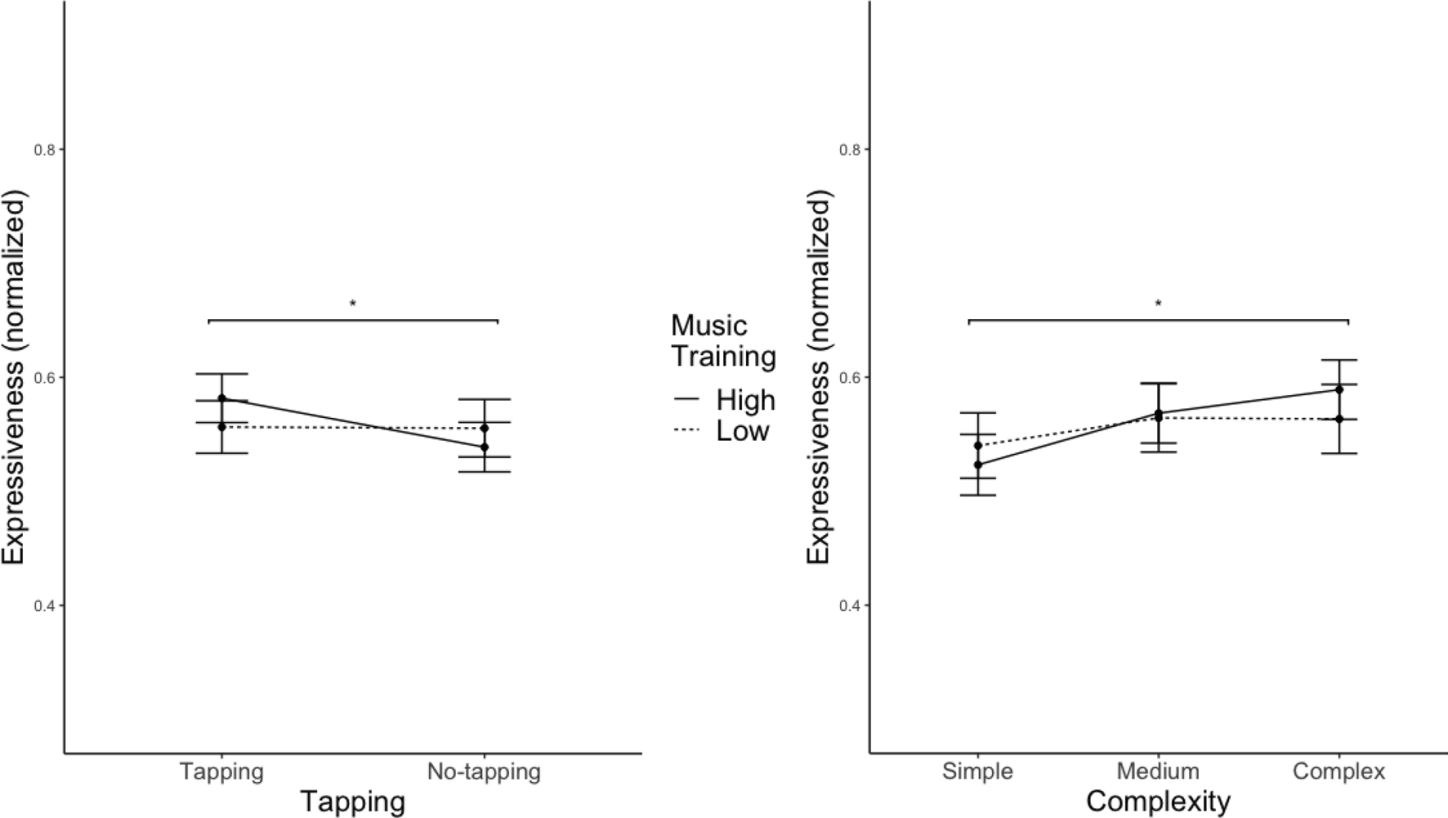
Line plots of the interaction effects between training and tapping (left), as well as between training and complexity (right) on Expressiveness. The whiskers represent standard errors. **p < 0.01, *p < 0.05. Significance indicators are of the same line type as the corresponding groups (i.e. musical training)

Significant main effects of tempo and training were found, whereas there was no significant effect of complexity and tapping (see Table 1, for the full table, please refer to Table S2). Faster tempo is related to higher perceived Expressiveness, as post-hoc analyses with Tukey corrections revealed significant differences between slow and fast, slow and medium, as well as medium and fast conditions. Expressiveness ratings were the highest with fast tempo, followed by medium, and lowest with the slow tempo (see Table 3). The model suggested significant interaction effects between training and tapping, as well as between training and complexity (see Table 1). Post-hoc comparisons with Tukey correction showed that the effect of tapping was only significant for highly musically trained participants (see Fig. 5, left panel): they perceived the performances as more expressive when tapping than when not tapping. Furthermore, the effect of complexity was only significant for highly musically trained participants (see Fig. 5, right panel): the more complex the stimuli, the more expressive they were perceived.

### Influence of tapping speed and stability

For the tapping trials, LMMs were conducted in order to analyse specific effects of tapping in terms of speed and stability. This part of analyses focused on the effects of tapping speed, stability, and their interactions with training, tempo, and rhythmic complexity on DE, PoT, and Expressiveness.

### Duration estimation

**Fig. 6:**
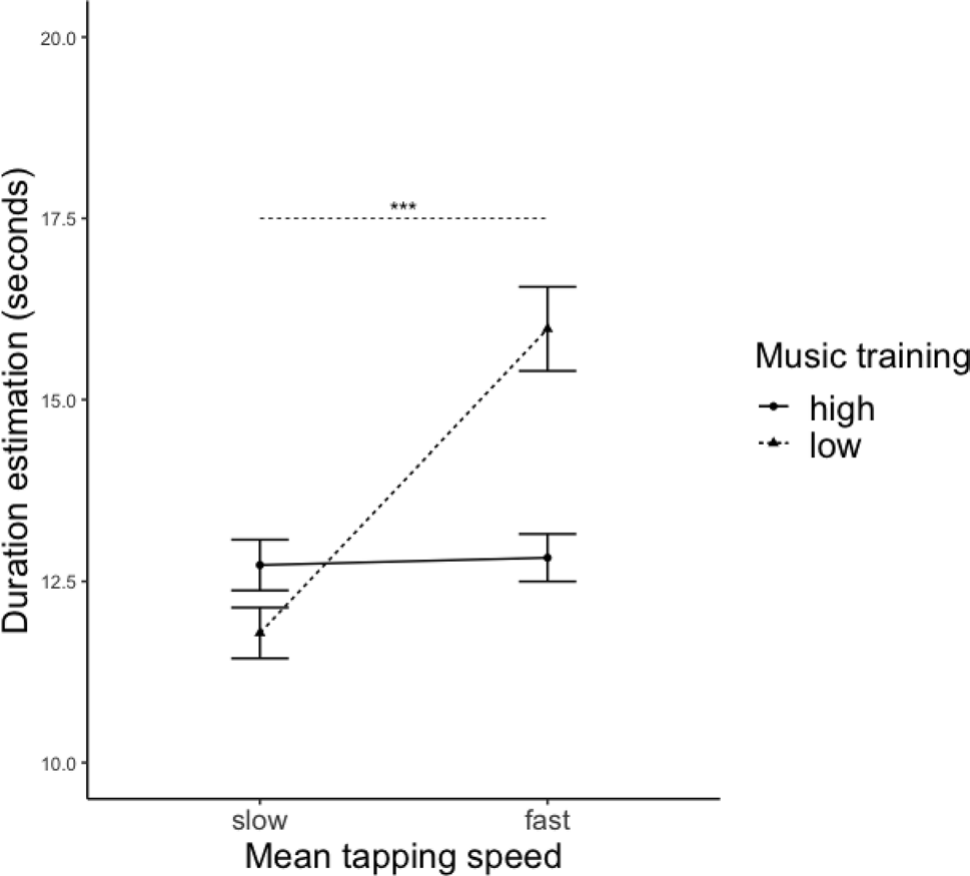
Line plots of the interaction effects between training and tapping speed on duration estimation. The whiskers represents standard errors. **p < 0.01, *p < 0.05. Significance indicators are of the same line type as the corresponding groups (i.e. musical training)

Significant main effects of tapping speed, stimuli tempo, and music training were found, while there was no main effect of tapping stability or stimuli complexity (see Table 4, for the full table, please refer to Table S4). However, post-hoc analyses with Tukey corrections revealed no significant differences between slow and fast, slow and medium, as well as medium and fast conditions (see Table 6). A significant interaction effect between tapping speed and training on duration estimation was found. Post-hoc analyses suggest that less musically trained participants (MT score below group median) were affected by their own tapping speed: the faster they tapped, the longer they estimated the duration; whereas participants with more musical training (MT score above group median) were not affected (see Table 5 and Fig. 6).

**Table 4:** Summary of the mixed linear model analysis for tapping trials only

DV	Variable	*B*	SE B	*t*	*p*
Duration estimation	${\beta }_{IT{I}_{mean}}$	− 5.15	1.93	− 2.66	0.008**
	${\beta }_{Tempo}$	− 0.67	0.35	− 1.93	0.054*
	${\beta }_{MT}$	− 8.38	3.00	− 2.79	0.006*
	${\beta }_{IT{I}_{mean}:MT}$	4.98	2.59	1.93	0.055*
Passage of time	${\beta }_{IT{I}_{CV}}$	0.86	0.33	2.57	0.010*
	${\beta }_{Tempo}$	− 0.08	0.02	− 5.59	< 0.001***
	${\beta }_{ITI mean:CV}$	− 1.05	0.45	− 2.35	0.018*
Expressiveness	${\beta }_{Tempo}$	− 0.12	0.02	− 7.70	< 0.001***

For the full table, please refer to Table S4

*ITI*_*mean*_ normalized tapping speed, *ITI*_*CV*_ tapping stability, *Comp* complexity, *MT* normalized music training scores. For coding of variables, see Table 1

***p < .001, **p < .01, *p < .05

**Table 5:** Summary of the post-hoc comparison with Tukey correction based on significant interactions from Table 4

DV	Condition1	Condition2	Differences of estimate	SE of difference	*t*	*p*
Duration estimation	High MT	High ITI_mean_ − Low ITI_mean_	0.57	0.42	1.36	0.528
	Low MT	High ITI_mean_ − Low ITI_mean_	1.76	0.46	3.80	< 0.001***
Passage of time	High ITI_mean_	High ITI_CV_ − Low ITI_CV_	− 0.004	0.02	− 0.22	0.996
	Low ITI_mean_	High ITI_CV_ − Low ITI_CV_	− 0.009	0.02	− 0.50	0.959

For coding of variables, see Table 1

*ITI*_*mean*_ normalized tapping speed, *ITI*_*CV*_ tapping stability, *MT* normalized music training scores

***p < 0.001, **p < 0.01, *p < 0.05

### Passage of time

Significant main effects of tapping stability and tempo on PoT were found, whereas there were no main effects of complexity and training (see Table 4, for the full table, please refer to Table S4). Post-hoc analyses with Tukey corrections revealed significant differences between slow and fast, slow and medium, as well as medium and fast conditions (see Table 6). PoT was perceived the fastest with fast tempo, followed by medium tempo, and was perceived the slowest with slow tempo. Furthermore, a significant interaction between tapping stability and tapping speed was found. Post-hoc comparisons with Tukey correction suggested that the effect of mean tapping speed did not differ in relation to tapping stability (see Table 5).

**Table 6:** Summary of the post-hoc comparison with Tukey correction based on significant main effects from Table 4

DV	Condition1	Condition2	Differences of estimate	SE of difference	*t*	*p*
Duration estimation	Slow	Fast	0.44	0.31	1.41	0.387
	Slow	Medium	0.71	0.29	2.45	0.123
	Medium	Fast	− 0.27	0.28	− 0.94	0.645
Passage of time	Slow	Fast	− 0.22	0.01	15.76	< 0.001***
	Slow	Medium	− 0.12	0.01	8.64	0.003**
	Medium	Fast	− 0.10	0.01	7.26	0.004**
Expressiveness	Slow	Fast	− 0.25	0.02	11.17	< 0.001***
	Slow	Medium	− 0.17	0.02	7.80	0.003**
	Medium	Fast	− 0.08	0.02	3.42	0.067

For coding of variables, see Table 1

*T* tapping, *NT* no-tapping

***p < 0.001, **p < 0.01, *p < 0.05

### Expressiveness

**Fig. 7:**
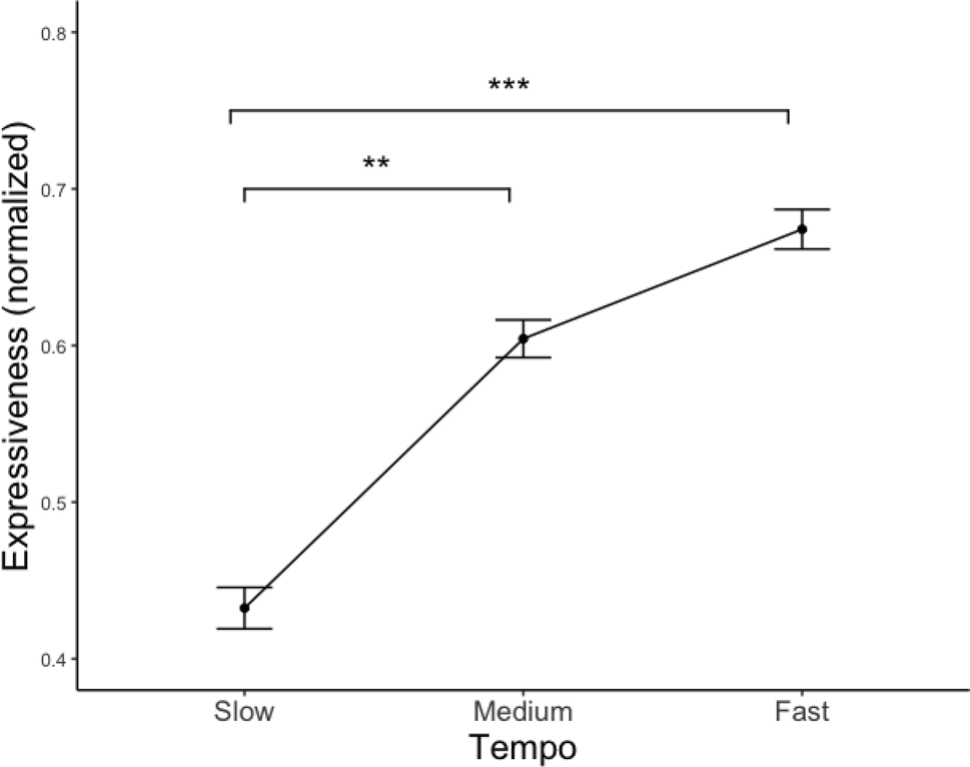
Line plot of normalized Expressiveness grouped by performance tempo. The whiskers represent the standard errors. ***p < 0.001, **p < 0.01, *p < 0.05

A significant main effect of stimuli tempo was found: the faster the tempo, the more expressive the performance was perceived (see Fig. 7, Table 4). Post-hoc analyses with Tukey corrections revealed significant differences between slow and fast, as well as slow and medium tempo conditions. Expressiveness was perceived to be higher with fast than slow tempo, and higher with medium than with slow tempo. However, no main effects of tapping speed, stability, complexity, training, as well as of the two-way interactions among the variables were found.

## Discussion

In this study, we performed an online experiment aiming at comparing participants’ perception of time and expressiveness when tapping and not tapping to performances of a professional drummer. Participants were required to judge Duration (DE), Passage of Time (PoT), and Expressiveness in both conditions. The results suggested that time judgments and perceived expressiveness are related to motor involvement, i.e. tapping versus not tapping. Musical training also mediated the effects of motor involvement on duration estimation as well as perceived expressiveness. In addition, tempo and complexity as musical attributes have contributed to the temporal judgments.

### Tapping versus no tapping

Regarding the effect of tapping, we found that the tapping trials were perceived shorter in durations than no-tapping trials at slow and medium tempi. The effect of tapping is thus partially in line with our hypothesis (H1) and previous findings, such that tapping may have reduced the attentional resources allocated to the passing of time ( Hammerschmidt & Wöllner, 2020; Wöllner & Hammerschmidt, 2021). As participants in the current study focused on the tapping task, they attended less to the timing task than when they were not tapping. This may have led to fewer temporal units registered in the internal clock system (Block et al., 2010), and thus shorter durations were perceived. However, the effect was only present with slow and medium tempi. One possibility is that, when tapping to fast-paced stimuli, it could be more difficult to maintain an isochronous beat which requires higher tapping stability than with slow and medium tempi, in line with audio-visual thresholds for stable tapping (Repp, 2003). In this case, participants were possibly more engaged with the timing tasks with fast tempo, which explains the absence of the tapping effect.

As an additional finding of our study, tapping had an effect on expressiveness under specific conditions: for the musically trained group, tapping trials were perceived to be more expressive than no-tapping trials. As musicians are more familiar with sensorimotor synchronisation due to training in music performance compared to non-musicians (Nguyen et al., 2022), tapping could more likely have induced higher perceived expressiveness than the no-tapping condition for them.

### Tempo and complexity

Our study found no significant main effect of tempo on DE and PoT when both tapping- and no-tapping trials were included in the analysis. However, with only the tapping trials, tempo has a main effect on PoT: the faster the tempo, the faster the PoT. The finding partially supports our hypothesis H2. The presence of a tempo effect on PoT but not on DE has been seen in previous research. In a study where participants were asked to judge seconds-range and minutes-range durations in daily life, changes in the perceived PoT but not DE were found only for the seconds-range durations (Droit-Volet et al., 2017). This might explain our findings as we adopted 15-s stimuli. Droit-Volet and colleagues (2017) argued that duration estimation required conscious attention to time, which was less emergent in seconds-range timeframes than in minutes-range. With regard to tempo and duration estimation, the current findings are in line with a duration estimation task using tempo-shifted disco music (Hammerschmidt et al., 2021a, 2021b). Tempo differences between 105 and 125 BPM did not elicit changes in participants’ perceived durations, suggesting a low sensitivity towards internal clock speed changes in the duration task.

The finding of a tempo effect on PoT only with the tapping trials highlights the role of motor involvement in time perception. This effect aligns with our hypothesis (H1) that tapping trials should pass faster than no-tapping trials, and is supported by past studies, where tapping as an additional task to the timing judgments has increased cognitive load, therefore, diverted the attention resources allocated to the timing tasks (Wöllner & Hammerschmidt, 2021). Similarly, in other prospective timing paradigms, additional tasks that entail higher cognitive load were linked to duration underestimation, as unattended temporal pulses could not register on the accumulator-counter device (Block et al., 2010).

Our finding that high complexity led to faster PoT does is in contrast to hypothesis H2 that complex music should lead to slower PoT. Previous findings revealed that participants judged complex audiovisual stimuli to last longer, indicating increases in the internal clock speed (Bueno et al., 2002; Schiffman & Bobko, 1974). One explanation could be that they adopted grouping strategies with more complex stimuli, as the musical structure exceeded one’s ability to follow note by note. According to the grouping principle proposed by Lerdahl and Jackendoff (1983), listeners could segment a musical excerpt based on its hierarchical structure of the notes. It has also been pointed out that the variations in listeners’ grouping strategies might be due to shifts in attention (Clarke & Krumhansl, 1990). Considering this possibility, participants in the current study may have attended to musical accents of higher metrical levels (i.e. half- or whole-note level instead of eighth- or quarter-note level) with more complex stimuli as a grouping strategy, resulting in fewer temporal units, as the internal clock was entrained to a slower pulse and faster passage of time. This is in line with findings that attention shifts to higher metrical structures led to duration underestimation, indicating a slower clock speed compared to lower metrical structures (Hammerschmidt & Wöllner, 2020). By potentially entraining the speed of the internal clock to a higher metrical level, participants might not necessarily be affected by the increased event density in complex stimuli.

For both tapping and no-tapping trials, faster tempo was related to higher perceived expressiveness. The association between performance tempo and emotional expressiveness is in line with our hypothesis (H2) that faster stimuli are perceived to be more expressive. Tempo has been regarded as one of the most important factors that facilitates the expression of emotions with music (Juslin & Madison, 1999; Juslin et al., 2001). Faster tempo has been linked to higher felt emotional arousal (Droit-Volet et al., 2013), while music perceived high in arousal level has been associated with high expressiveness (Fernández-Sotos et al., 2016). The current study is further in line with Allingham et al.’s (2021) research, as they have found an association between increases in movement speed and a rise in perceived expressiveness.

### Musical training

For musically trained participants, the more complex the rhythms, the higher they perceived the expressiveness. The effect was absent for less trained participants. Our observation is partially in line with past research, in which non-musicians, non-drummer musicians, and drummers rated the expressiveness of drumming performances differently by allocating different weights on musical tempo, presentation modalities, and genres (Di Mauro et al., 2018). In this study, musically trained and less trained participants were both sensitive to the musical emotions expressed, whereas the trained group focused more on the technical aspects of the performances such as complexity when they judged emotional expressiveness.

Given the overall effects of motor involvement in time perception, we found that tapping speed affected duration estimation of the less musically trained group, but not the highly trained group. This partially supports our hypothesis (H3), that musical training should be associated with higher accuracy in DE and PoT. The finding highlights the role of music training in the timing and temporal judgments. The better performance among the trained group could be due to increased sensitivity towards the underlying rhythmic structure. In this way, the musically trained group (1) may have perceived the beats to be more salient than the less trained group, (2) considering that synchronizing with a beat is common practice in music training, they tapped more accurately to the drum beats, which facilitated their timing, and (3) even if there was variation in their tapping behaviour, they were less affected by it. In turn, they could better register the temporal units in the pacemaker-counter device, and estimated the stimuli duration consistently regardless of their own tapping speed. This is in line with previous studies, in which musically trained individuals outperformed the less trained group in a number of timing tasks by showing higher accuracy in duration estimation and synchronization with beats (Panagiotidi & Samartzi, 2012; Rammsayer & Altenmüller, 2006; Repp, 2010).

### Tapping speed

In addition, the effect of tapping speed on duration estimation for the less trained group also partially confirmed hypothesis H4 that faster tapping speed should lead to duration overestimation. The temporal entrainment effect (Barnes et al., 2000), describing the variation of temporal pulse emission following the rhythms of external events, offers a possible explanation in this context. As participants changed their tapping speed, the process also elicited a changing internal clock speed by adjusting the temporal pulses emitted by the internal clock accordingly. The faster they tap, the more temporal units were accumulated, the longer a duration might be perceived. Furthermore, tapping could reinforce the temporal entrainment effect by increasing the salience of beats and drawing more attentional resources. In this way, each tap serves as a clear reference between short intervals of time that facilitate the accumulation of clock “ticks” on the counter device. Furthermore, evidence suggests that tapping can be effectively associated with the metrical levels that are registered by the variation of force (Benedetto & Baud-Bovy, 2021). The finding supports the possibility that tapping can be used to annotate how people perceive the rhythms, and consequently the perception of time passed (Hammerschmidt & Wöllner, 2020; Wöllner & Hammerschmidt, 2021).

### Limitations

A potential limitation of the current study is the sequence of tasks, especially for duration estimation and passage of time. The current study presented the PoT judgments after the duration estimation task for all participants, with both questions appearing on the webpage immediately after the stimulus. Estimation of duration may thus have affected their subsequent PoT judgments. According to the internal clock theory (pacemaker-counter mechanism), the judgment of the current time is based on the comparison of the temporal units registered to a reference duration, i.e. how fast a given amount of time should normally pass and how long it should feel, which is highly subjective (Wearden, 2015). In the current study, it is unclear what the reference duration was for each participant. Should a participant judge the stimulus to be, for example, 60 s compared to 20 s (in clock time), their PoT judgment may be under the influence of prior duration judgment. Consequently, the immediate effect of our variables such as tapping may be moderated by the duration judgments that lies between the stimulus and the PoT task. There is evidence that for an association (Droit-Volet et al., 2015) as well as a disassociation (Droit-Volet & Wearden, 2016; Droit-Volet et al., 2017) between duration estimation and PoT (Droit-Volet & Martinelli, 2023 in press). Future studies should nevertheless attempt to capture the nuances in both PoT and DE.

Another limitation of the study is that, by providing three types of duration (15 s for the main trials, 8 s and 30 s for the catch trials), there is the possibility that participants might have become consciously aware of the test durations and responded accordingly in a categorical way. Although variations both within each participant’s duration estimation and across the group have been observed, future studies should control for this potential issue by asking participant whether they had been aware of durations and other potential control variables during their responses and had been affected accordingly.

## Conclusion

The current study investigated perceived time and expressiveness when tapping and not tapping to drumming performances that varied in tempo and complexity. Our main finding that time passed faster and felt shorter in duration (at slow and medium tempo) when tapping to drumbeats compared to when not tapping, could shed light on our time experiences in everyday scenarios such as when people are moving to music rather than passively listening to it. While motor involvement and focus of attention have clearly influenced the findings in relation to embodiment and internal clock models, more specific effects of synchronisation stability and speed call for further research.

## Data Availability

Stimuli and data are accessible to the editors and reviewers.
